# Scientific Items

**Published:** 1887-11

**Authors:** 


### SCIENTIFIC ITEMS.

**Theine.**—One of the most remarkable of all local agencies, so remarks the *Dental Review*, which has been before the profession, is theine, the alkaloid of Chinese tea. Attention was first drawn to this agent by Dr. Mays, of Philadelphia, in an article on "The Physiological and Therapeutic Action of Caffeine, Theine, and Guarine."

It was shown from experiments made on frogs that theine paralyzes the nerves of sensation, and this impairment of sensibility proceeds from the centre to the periphery, and not like that of brucine, from the periphery to the centre.

Among the extraordinary features of theine is its localized anæsthesia below the seat of injection. There can be no question about its general absorption; but there has occurred no evidence in the experience of its use to show that it produces any systematic disturbance, even in maximum doses. Its absorption and distribution by the blood probably play a secondary role, for it seems to exert its influence on the trunk of the nerve, at the point of its injection, and then to disiribute itself downward along its course in spite of these functions. This localized action of theine naturally gives it an immense advantage over morphine, atropine chloral, and other agents of that class, which principally act by intoxicating the central nervous system.

The leaf of Chinese tea has formed a domestic drink for many centuries, and its alkaloid, theine, has been known for nearly three-score years, yet no one suspected that it contained an analgesic action more remarkable than that of cocaine.—*National Druggist.*

**Electriffed Ice Cream.**—Dr. Geo. S. Hull, of Chambersburg, Pa., has found by actual experiment that electricity is generated in ice cream by the friction of the zinc paddles on the contents of the freezer. He has experimented with various mixtures, and found that the presence of fruit or other acids increased the electric action. The doctor concludes that the case of ice cream poisoning come from the zinc and not ptomaines or tyrotoxicon. The doctor is zealously pushing his investigations, and we look for further developments.—*National Druggist*, Aug. 15, 1887.

**Something New About Bees.**—At a recent meeting of the Royal Microscopical Society, Mr. F. R. Cheshire called attention to some specimens of bees, known as "fertile workers." It was generally well known that in the beehive all the eggs were usually laid by the queen, and in her absence no ovipositing occurs until they have taken some of the eggs remaining in the hive, and by a special feeding of the larvæ have been able to produce fresh queens. If, however, it should happen that in a hive which has lost its queen there are not eggs available for this purpose, it was found that some of the workers under some special circumstances, which could not be very clearly explained, became capable of laying eggs produced drones only. These bees were known as fertile workers, and though there could be no doubt as to their frequent existence, they were very difficult to catch, owing to their being the same in appearance as the ordinary workers. He now exhibited two of these fertile workers having the ovaries drawn out of the bodies and attached to the stings and abdominal plates, so as to show that they really were workers. There was a remarkable peculiarity to be observed in connection with the ovarian tubes of these insects—every ordinary worker possessed an undeveloped ovary which it was difficult both to detect and dissect; but when under the influence of some stimilus the worker became fertile, a number of points began to appear in the tubes which afterward became developed, and it would seem that the eggs were developed in alternation, an examination of the tubes showing them to contain developed eggs alternating with others in an undeveloped condition, and of which some very curious instances were seen in the specimens before the meeting.—*Scientific American.*

**Interesting Cure of Insanity.**—An interesting instance of fighting insanity by insanity has recently been noticed among the Blackwell's Island patients. Two lunatics had been received who were disposed to commit suicide. In addition each possessed a special delusion, one to the effect that he was a cow, the other that his head was an iron ball, and was to be rolled along the floor. They carried these beliefs into action, one striking his head against the padded walls of his cell, the other rolling his head, and, of course, his body with it, along the floor. The two patients were placed together, and each was privately informed of the other's weakness and warned to watch his companion to prevent him taking his own life. Thus each had a charge in the other. Their vigilance was unceasing. Each supposed himself perfectly sane, and this belief was returned by considerable scorn for the other's weakness of intellect and accompanying delusions. Gradually under the influence of this treatment the patients were observed to improve. To have their attention centered on definite duty and on objects external to themselves proved a tonic for their diseased minds, and gradually a complete cure was affected, and they received their discharge from the asylum.—*Scientific American.*

**The Skeleton in the Cupboard.**—Take a bottle of phosphorized oil, labelled "The Phantom Light," and a small brush, packed in car-board box. Get a large sheet of paper, and then, with the aid of the brush dipped into the phantom light, roughly sketch the outline of the human skeleton; then attach it to the wall in an empty cupboard, shut the door, take your friends into the room in which the cupboard is (the room being quite dark), and ask one of them to go to the cupboard. He will, no doubt, run back greatly alarmed. Other devices may be adopted at the will of the operator.—*Scientific American.*

